# TURead: An eye movement dataset of Turkish reading

**DOI:** 10.3758/s13428-023-02120-6

**Published:** 2023-07-05

**Authors:** Cengiz Acartürk, Ayşegül Özkan, Tuğçe Nur Pekçetin, Zuhal Ormanoğlu, Bilal Kırkıcı

**Affiliations:** 1 Cognitive Science Department, Jagiellonian University, Kraków, Poland; 2 Cognitive Science Department, Middle East Technical University, Çankaya/Ankara, Turkey; 3 Department of Foreign Language Education, Middle East Technical University, Çankaya/Ankara, Turkey

**Keywords:** Eye movements, Oculomotor control, Silent reading, Oral reading, Turkish

## Abstract

In this study, we present TURead, an eye movement dataset of silent and oral sentence reading in Turkish, an agglutinative language with a shallow orthography understudied in reading research. TURead provides empirical data to investigate the relationship between morphology and oculomotor control. We employ a target-word approach in which target words are manipulated by word length and by the addition of two commonly used suffixes in Turkish. The dataset contains well-established eye movement variables; prelexical characteristics such as vowel harmony and bigram-trigram frequencies and word features, such as word length, predictability, frequency, eye voice span measures, Cloze test scores of the root word and suffix predictabilities, as well as the scores obtained from two working memory tests. Our findings on fixation parameters and word characteristics are in line with the patterns reported in the relevant literature.

## Introduction

The study of reading requires the investigation of perceptual and cognitive processes at multiple levels, including oculomotor control, word identification, sentential level processes, and discourse comprehension. Over the past two decades, eye movement control models and analysis methods have been developed to investigate the relationship between word recognition dynamics and eye movements during text reading, aiming to explain directly unobservable reading processes such as word recognition in terms of observable phenomena, mainly eye movements. Numerous features of words are used as variables, the most popular being word frequency, word length, and the predictability of words in sentential contexts. Depending on the stimulus design, these features (e.g., word frequency and length) are calculated using general purpose corpora and massive data collection sessions (e.g., sentential predictability). Dependent variables include numerous oculomotor parameters, such as single fixation duration on target words, pre-target words, post-target words, fixation counts, and regressions within or across word boundaries, e.g., Inhoff et al. (2011); Kliegl et al. (2006); Kliegl (2007); Laubrock & Kliegl (2015); Pollatsek et al. (2000); Hyönä & Pollatsek (1998); Järvilehto et al. (2009); Yan et al. (2014); Deutsch & Rayner (1999); Paterson et al. (2015); Özkan et al. (2021)); see Rayner (1998, 2009a, 2009b); Rayner et al. (2012) for reviews.

As another crucial aspect in reading research, a diverse set of experimental paradigms have been used to study the role of sound coding in skilled reading, including masked phonological priming, articulatory suppression, auditory input distraction, electromyography recordings of articulatory muscles, and the gaze-contingent boundary paradigm, which often accompany tasks such as naming, lexical decision, semantic categorization, and sentence or text reading. The findings obtained provide supporting evidence for the presence of sound coding in the reading process. There is an ongoing debate on the possible impact of sound encoding on eye movements in reading. Such an impact may be realized as early involvement of phonological processes in lexical access, or the retention of words can be realized as phonological representations during post-lexical integration (Slowiaczek & Clifton, 1980; Van Orden, 1987; Guerrero, 2005; Inhoff et al., 2004; Eiter & Inhoff, 2010; Ashby et al., 2006; Frost, 1998, 2005; Coltheart et al., 2001; Rastle and Brysbaert, 2006; Acartürk et al., 2017; Leinenger, 2014). Previous research, which is based mainly on empirical investigations, shows that direct auditory input registration (Inhoff et al., 2004) and its combination with articulatory suppression as a secondary task (Slowiaczek & Clifton, 1980; Eiter & Inhoff, 2010) influence the duration of subsequent fixation and comprehension of the text. However, studies focusing on Eye Voice Span (EVS) in reading aloud have indicated a dynamic modulation of EVS, keeping a uniform distance between the eyes and the voice, thus implying the retention of a manageable number of items in working memory; see Inhoff et al. (2011); Laubrock & Kliegl (2015); Leinenger (2014) for reviews of the role of sound coding in post-lexical processing in reading.

Eye movement datasets usually present the characteristics of words and a set of eye movement variables concerning the words in a text and, thereby, provide eye movement data for analyses. Recently, eye movement corpora for numerous languages have emerged (Schilling et al., 1998; Kennedy et al., 2003, 2013; Kliegl et al., 2004; Laurinavichyute et al., 2019; Luke & Christianson, 2018; Pan et al., 2021), some of which have been established on multiple dimensions, such as monolingual and bilingual reading (Cop et al., 2017), cross-linguistic multilingual reading (Siegelman et al., 2022), and reading development in children in both silent and oral modalities (Vorstius et al., 2014). The existing eye movement datasets vastly differ in their material selection methodologies. Some employ an experimental approach, in which a set of selected target words is manipulated (Kliegl et al., 2004; Laurinavichyute et al., 2019; Vorstius et al., 2014), while others employ a corpus-analytical approach, in which participants read a set of sentences without any manipulation of target words (Kennedy et al., 2003, 2013). Another aspect in which the existing eye movement datasets differ is the selection of the variables investigated. For example, some available datasets include predictability norms besides word frequency and length (Kennedy et al., 2003, 2013; Kliegl et al., 2004; Laurinavichyute et al., 2019; Luke & Christianson, 2018; Pan et al., 2021), while others only provide the eye movement data (Siegelman et al., 2022). Another aspect is that while most eye movement corpora cover silent reading, some oral reading data is also available with a specific focus on Eye Voice Span (Inhoff et al., 2011; Laubrock & Kliegl, 2015).

The present study presents TURead, an eye movement dataset of reading in Turkish, a largely understudied language in reading research. A small dataset of eye movements in Turkish was recently made available for silent reading, established using a corpus analytical approach (Özkan et al., 2021). TURead differs from this available dataset in several dimensions. TURead assumes an experimental approach in which target words were manipulated based on word length, frequency, and the number of suffixes. The main body of TURead includes lexical and prelexical characteristics of the target words, their predictability scores, and specific measures for oral reading. TURead aims to provide empirical data to facilitate further investigations of the reading characteristics of Turkish, an agglutinating language with rich morphology and shallow orthography. These characteristics of Turkish make it particularly suitable for studying early phonological processing, word frequency and length effects, and morphological complexity, which may be conceived as the primary components of the cognitive processes involved in reading.

Regarding the investigation of word identification processes in Turkish reading, in a study of sentential pseudoword reading in Turkish, several measures of letter frequency showed significant effects on measures of eye movement (Acartürk et al., 2017). Statistically significant effects on fixation duration were obtained for word center consonant collocation frequency and word boundary frequency, in addition to a significant interaction of vowel harmony collocation frequency (reflecting the vowel harmony rules that restrict vowel sequences in Turkish words) and word boundary collocation frequency. The observed effects were interpreted as instances of the impact of phonotactics on Turkish reading, since the stimuli consisted of pseudowords. Therefore, TURead was designed to include the prelexical characteristics of the target words to address the possible impact of phonotactics in Turkish.

In TURead, we included EVS measures, in addition to silent reading measures, to improve their potential to contribute to the study of the influence of phonological representations of words in reading. We also included the results from two memory tests (a Corsi Block test and a digit span test). A possible use of the results of the memory test is to investigate the relationship between the retention of items in the working memory and the reading process (for example, an analysis of the influence of the working memory scores on EVS as the number of words, Özkan 2020).

TURead includes an additional set of variables, mainly novel for reading research. One is suffix-level predictability values, and the other is familiarity ratings for target words. The former has the potential to be used in the analysis of morphological complexity, whereas the latter can be used to investigate early phonological processing within specific theoretical frameworks, such as the dual-route hypothesis, which assumes a direct lexical access route for words and an indirect access route through prelexical grapheme-to-phoneme rules for novel words (Coltheart et al., 2001). Finally, TURead can also be used as a new benchmark for future research on Turkish reading. In the following section, we present the methodology behind TURead.

## Methodology

### Participants

A total of 215 participants (*M* = 22.72, *SD* = 2.61 years old; 102 females) participated in the experiment for monetary compensation of approximately 5 US Dollars. Each participant signed an informed consent form and completed a demographic data form prior to the eye movement recording session. We excluded data from 15 participants, since (i) the native language of one participant was not Turkish, (ii) eight participants identified themselves as bilingual, (iii) two participants reported having dyslexia, (iv) two participants used contact lenses during the experiment (no participant had corrected vision with glasses) and (v) two participants read 50% of the stimuli text twice due to technical problems (total data loss 6.9%; *M* = 23.13 years old, *SD* = 2.36; seven females). An inspection of the eye movement data recorded revealed that the data of the other four participants were not eligible due to technical problems, such as electricity supply problems during the recording session (data loss 1.9%; *M* = 21.00 years old, *SD* = 2.08; two females). As a consequence, the data collected from 196 participants were included in TURead (91.2% of 215 participants; *M* = 22.72 years old, *SD* = 2.64; 93 females).

### Materials

TURead consists of 192 short texts, each composed of 1-3 sentences (s). Each text includes a target word designed for the purpose of the study. The target words were selected from the BOUN web corpus according to their stem frequencies and lengths. The BOUN web corpus includes 1,337,898 distinct words (types) and 383,224,629 word tokens (Sak et al., 2008). The surface frequency of a word was calculated in terms of word tokens such that the surface count was the sum of the occurrences of the exact form of the word in the corpus.

Two groups of target words were selected from the BOUN corpus based on their stem surface frequencies: *low frequency* words and *high frequency* words. The cut-off point for stem surface frequencies was 0.75 (frequency per million), which was the mean of the BOUN corpus (*SD* = 35.50).^1^ As for word length, TURead included *short* target words and *long* target words. The stems of the short target words consisted of four letters (for example, *masa*, ‘table’), while the stems of the long target words consisted of ten letters (e.g., *bilgisayar*, ‘computer’). Consequently, the target word set had four conditions based on the combination of stem length and surface stem frequency (henceforth, conditions): Short-Infrequent (SI) words, Long-Infrequent (LI) words, Short- Frequent (SF) words, and Long-Frequent (LF) target words.

There were 16 words per condition. The stimuli (that is, the texts) also included suffixed forms of the target words, which bore the allomorphs of the Turkish locative marker *-DA* (*-de* / *-da* / *-te* / *-ta*), and of *-DAki*,^2^ the combination of the locative marker and the suffix -ki (*-deki* / *-daki* / *-teki* / *-taki*). The selected suffixes are among the most frequently used in Turkish, as revealed by an analysis of suffix frequencies in the corpus. In total, the target word set consisted of 192 words.

The four word conditions were constructed such that the characteristics of the target words (i.e., stem length and frequency per million) were homogeneous within each condition, as desired for the validity of the design. In other words, the mean frequency per million values were not different between short and long words within each frequency condition for stems, one-suffix words, and two-suffix words (e.g., the mean stem frequency per million values of short words were not significantly different from that of long words among frequent words). The mean surface frequency values (per million) of the stem and suffixed versions of the target words, together with the ANOVA results, are presented in Table 1.

**Table 1:** Mean surface frequency values of target words by condition and ANOVA results of the difference between mean frequency values between length conditions

	Frequent Words	Infrequent Words
Surface frequency per million		
	**Stems**	
Short Words	26.02 (18.93)	0.07 (0.09)
Long Words	47.63 (58.45)	0.14 (0.17)
	*F*(1,30) = 1.98, *p* $>.05$	*F*(1,30) = 2.27, *p* $>.05$
One-suffix words		
Short Words	7.73 (14.72)	0.001 (0.002)
Long Words	4.22 (7.58)	0.002 (0.004)
	*F*(1,30) = 0.72, *p* $>.05$	*F*(1,30) = 0.64, *p* $>.05$
Two-suffix words		
Short Words	1.10 (2.19)	0.0003 (0.001)
Long Words	0.22 (0.33)	0.0003 (0.001)
	*F*(1,30) = 2.52, *p* $>.05$	*F*(1,30) = 0.00, *p* $>.05$

Values in parentheses represent standard deviations

The texts consisted of 1-3 sentences. The sentences within the texts were selected from a set of sources, including the BOUN Corpus (Sak et al., 2008), the METU Turkish Corpus (Say et al., 2002), and the Turkish National Corpus (Aksan et al., 2012). Due to the agglutinating structure of Turkish, it was difficult to find suffixed forms of infrequent words within the aforementioned sources. In such cases, the sentences were retrieved from publicly available sources (e.g., search engine results) or a synonym was used in place of a target word in a sentence. This methodology allowed us to use publicly available texts instead of generating sentences on purpose, thus improving the ecological validity of the experiment. In addition to the stimuli texts, four paragraphs were used as filler material. The paragraphs were excerpted from a novel (Bıçakçı, 2006). Stimuli texts are publicly available in the online repository.^3^

In the resulting stimuli, neither the number of words (*M* = 15.33, *SD* = 2.88 words) in each text nor the number of characters (*M* = 125.13, *SD* = 20.78 characters) were significantly different between the experimental conditions (*F*(3,188) = 1.00, *p* > 0.05, *F* (3, 188) = 2.20, *p* >.05, respectively). As for the number of characters in each line that included a target word, there were no significant differences between the four conditions (*M* =60.92, *SD* = 4.50, *F*(3,188) = 0.65, *p* > 0.05), see Table 2.

Another design principle applied during the development of TURead was that the target words were located approximately in the middle of a line. In other words, the number of characters to the left of a target word (*M* = 26.66, *SD* = 8.52) was close to the number of characters to the right (*M* = 25.77, *SD* = 7.93). The target words were also located approximately in the middle of the text. The character count from the onset of the text to the onset of the target word was *M* = 56.70 (*SD* = 27.85), and the character count from the end of the target word to the end of the text was *M* = 59.43 (*SD* = 22.93).

As briefly stated above, some of the stimuli texts consisted of more than one sentence (155 texts include a single sentence, 34 texts include two sentences, and three texts include three sentences). Orthographically, each text was presented on at least two lines (107 of 192 texts) and at most three lines (85 of 192 texts). There were at least two words between a target word and the onset or end of a sentence. Another principle of stimuli design was that sentences were selected from available corpora or public resources such that there was no punctuation mark around the target words. There were at least two words between the target word and the conjunction in case of the presence of a conjunction in a sentence. Finally, each text included only one target word. Each target word appeared in one single text and only once in a text. Hence, each target word and its suffixed forms appeared only once in the stimuli text set.

**Table 2:** Word and character counts of the stimuli texts

	Frequent Words	Infrequent Words
Word Count		
Short Words	15.73 (2.55)	15.48 (3.05)
Long Words	15.35 (2.82)	14.75 (3.05)
Character Count		
Short Words	124.65 (18.95)	121.00 (20.17)
Long Words	131.33 (20.99)	123.54 (22.12)

Values in parentheses represent standard deviations

### Apparatus

The eye movements of the participants were recorded using a monocular camera (right eye) embedded in an SR Research EyeLink 1000 eye tracker system with a tower mount, which has a recording frequency of 1000 Hz. The stimuli were presented on a 17-inch CRT monitor with 1024 x 768 resolution, with a VGA connection to a computer running at 3.0 GHz under the Windows XP operating system. The audio files were recorded for each text stimulus and the filler paragraphs using a compatible sound card (Creative Labs Sound Blaster Audigy 2 ZS). The participants were seated approximately 65 cm away from the display screen with their heads positioned on a forehead rest. The stimuli were presented using 18 pt monospace font (Courier New), each letter corresponding to 14.03 pixels, and approximately 0.46 degrees of visual angle. Since the experiment included oral reading blocks, only the forehead, but not the chin, was fixed with a chinrest to minimize head movements.

Each text was followed by a Yes/No comprehension question. The participants answered the comprehension questions using a Microsoft USB Sidewinder gamepad, and proceeded with the experiment after breaks, calibrations, and reading instructions. They were instructed to answer each question as *False* by using the back-left button or as *True* by using the back- right button. These instructions appeared below each question in parentheses.

### Design and procedure

The experimental stimuli were designed using the eye tracker manufacturer software, Experiment Builder version 1.10.1630. The recording session consisted of two parts each include two blocks, one silent reading block, and one oral reading block (that is, the reading modality), each lasting approximately 45 min. The experiment was conducted using a within-subject design. The reading modality of the texts and the order of the experiment blocks were counterbalanced by distributing 48 combinations (of the texts, conditions, the reading modality, and the order of the blocks) among the participants. The order of the texts within each block was also randomized. Consequently, each text was read both silently and aloud by different participants, and each participant read half of the stimuli texts silently and the remaining half of the stimuli aloud. Most of the participants completed both parts of the recording sessions on the same day (*N* = 213 of 215).

Each block consisted of a practice session (including four sample texts, two practice questions, and a filler paragraph) and a main reading session. The main reading sessions consisted of 48 stimuli texts in each block (cf. four target words x three suffix versions x four conditions). The entire recording session consisted of 192 stimuli texts and 48 true-or-false comprehension questions in total. The comprehension questions were prepared such that the correct answer to 94 of them was *False* and the remaining 98 required *True* as an answer. The participants correctly answered most of the questions (M = 88.84%, SD = 5.89%). There was a break after every 16 texts and between the blocks, summing up to ten breaks throughout the whole experimental session.

**Fig. 1:**
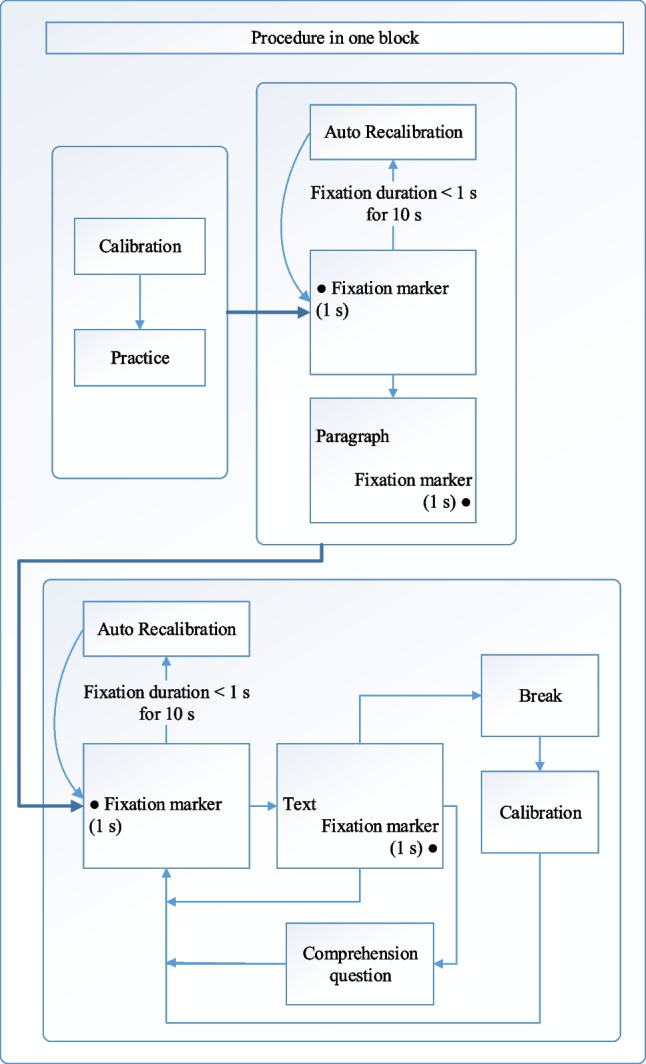
The procedure employed in each block. Each text was followed by a comprehension question, a fixation marker for the next screen, or a break

Instructions were presented to participants at the beginning and also between blocks for specific reading modalities. A nine-point standard calibration and validation was performed for the eye movement recordings. The calibration and validation procedures were renewed after each break. Participants were instructed to read the texts at their normal reading pace for comprehension, either silently or aloud, depending on the reading modality of the block. On the left of the screen, a gaze-contingent fixation marker (a circle with a diameter of 32 pixels) was displayed, on a blank screen, before the presentation of a stimulus on the screen. The coordinates of the fixation marker were px. 42 - px. 250 for the stimuli texts and px. 28 - px. 150 for the filler paragraphs (coordinate px. 0 - px. 0 defines the upper left corner of the screen). The non-visible IA (Interest Area) had a diameter of 150 pixels around the fixation marker. Following a fixation duration of the fixation marker of 1000 ms within the IA, the stimulus appeared on the screen with the first letter on the same coordinates as the coordinates of the fixation marker. If no fixation fell within the IA of the fixation marker for a duration of 1000 ms or longer for 10 s, an auto-calibration process was triggered for recalibration. Together with the texts, there was another fixation marker and an IA near the bottom right corner of the screen, which was the same size as the previous fixation marker. The coordinates were px. 982 - px. 700 for text stimuli and px. 981 - px. 715 for the filler paragraphs. The second fixation marker was also gaze-contingent. However, it was used to trigger the display of the next screen. Automatic recalibration was not triggered for the second fixation marker to avoid limiting the duration of the reading of the participants. If no fixation was detected for 1000 ms or longer in the IA of the fixation marker, the experimenter manually displayed the next screen using the keyboard of the host PC (that is, the computer that controls the eye tracker). This action started the automatic recalibration process. Figure 1 illustrates the procedure in one block. The procedure for the silent block and that of the oral block were identical.

For the analyses, each word was marked as an IA using the *Use Runtime Word Segment InterestArea* function of the Experiment Builder software. IAs for the fixation markers were constructed manually. The IA sets were then reconstructed to include the space before a word within the IA of that word. They were then used in Data Viewer version 3.2.1, the analysis software provided by the eye tracker manufacturer. Since the IAs for the fixation markers shown on the blank screen before the texts were used only during experiments for their gaze-contingent functions, the IAs for those fixation markers were removed from the IA sets for the analyses. However, the IAs for the fixation markers shown together with the texts were preserved in a new IA set to detect and eliminate rereading fixations.

### Memory and familiarity tests

Two memory tests were performed after the recording sessions (a Corsi Block test and a digit span test). Participants also completed an additional seven-point Likert scale familiarity test for target words. The raw scores of the memory tests were included in TURead with the variables names CORSI BLOCK SCORE and DIGIT SPAN SCORE. The mean values of the Corsi scores and the digit span scores are presented in Table 3.

**Table 3:** Mean scores and standard deviations of the digit span and Corsi tests (standard deviations presented in parentheses)

	Digit Span Test	Corsi Test
Score	6.99 (1.07)	5.73 (0.68)

The mean score of the Corsi test in the table presents data obtained from 195 participants due to a recording error in one participant

The familiarity test was administered after the recording session to prevent participants from seeing the target words prior to the experiment. Participants were instructed to score their levels of familiarity with the target words on a 7-point Likert scale (*1* for *I have never heard the word* and *7* for *I know the meaning of the word*). Our analyses revealed that the stem frequencies (per million) and the familiarity rating scores of the words were significantly correlated, *rs* =.856, *p* <.001. The raw scores from the familiarity rating task were included in TURead under the variable name FAMILIARITY RATING. The mean familiarity rating scores are presented in Table 4.

**Table 4:** Mean familiarity ratings for target words (standard deviations presented in parentheses)

	Infrequent Words	Frequent Words
Short Words	2.79 (1.52)	6.96 (0.02)
Long Words	4.02 (2.14)	6.95 (0.04)

### Predictability scores

The predictability scores for the target words were collected from 122 participants (mean age *M* = 24.01, *SD* = 4.26, ten participants did not report their year of birth; 82 females, one participant did not report gender), who did not participate in the main experimental recording sessions. A Cloze procedure was used to score sentential predictability (Taylor, 1953). Predictability scores were also collected for neighboring words of each target word (i.e., words n-1 and n+1) from a separate group of 70 participants, who made 35 predictions for each word (word n-1: *M* = 25, *SD* = 5.94 years old, two participants did not report their birth year; 21 females; word n+1: *M* = 25.6, *SD* = 5.07 years old, five participants did not report their year of birth; 25 females). Participants were asked to predict the following word (the target word n, the word n-1, or the word n+1) given the context in the part of the text prior to the target word. In addition to word predictability, two sets of suffix predictability data were collected from another group of participants, who also participated in neither the data recording sessions nor the word predictability scoring. A total of 110 participants were asked to predict the suffix of one-suffixed target words (*M* = 21.78, *SD* = 2.53 years old, seven participants did not report year of birth; 51 females, one participant did not report any gender), while 69 participants were asked to predict two suffixes of two-suffixed target words (*M* = 22.29, *SD* = 3.32 years old, three participants did not report birth year; 36 females) given the context in the part of the text prior to the suffix(es) of the target word, including the target word.

## The TURead dataset

In this section, we introduce the variables that provide general information in TURead, about the participants, the experimental blocks, the reading modality, and the stimuli texts (see Table 16 in the Appendix for general variables). We have also defined several variables to allow detailed analyses of the data. These are presented in Tables 17 and 18 in the Appendix. The full set of variables can be accessed in the online repository.

### Eye movement data inspection and cleansing

Eye movement data were inspected, cleansed, and corrected manually where necessary (e.g., in cases of regular offset errors), as described in this section. Eye movement measures were retrieved by Data Viewer analysis software.^4^

Manual inspection of gaze data revealed two types of calibration problems: (i) all fixations were above or below the lines (the *offset* problem), (ii) the fixations were upward or downward sloping. They were resolved by (1) selecting all fixations and moving them downward or upward, (2) selecting the fixations belonging to the same line and aligning them using the *Drift Correct* function of the Data Viewer, or (3) using a combination of (1) and (2). Fixations were moved only vertically when needed, and no fixations were moved horizontally (i.e., the coordinates of fixations along the X-axis were not updated) according to best practice in the literature (Holmqvist et al., 2011). When no solutions were applicable to a trial with calibration problems, that specific trial was removed from the analyses. Consequently, a total of 60 trials (of 37,632) were eliminated (0.16%). In 156 trials, the stimuli were read twice by the participant. These were also removed from the analyses (0.41%). The sum of the partial data loss was 216 trials (0.57%). Data loss statistics by elimination criteria are presented in Table 5.

**Table 5:** Eliminated data based on eye movement measures and articulation- related criteria

Criteria	Oral Reading (OR)	OR %	Silent Reading (SR)	SR %	Total	Total %
All data	18,816	-	18,816	-	37,632	-
Re-readings (due to technical problems)	24	0.13	132	0.70	156	0.41
Low-quality data	22	0.11	38	0.20	60	0.16
Valid data	18,770	99.76	18,646	99.1	37,416	99.43

No further data were eliminated, but the data were labeled to indicate the possibility of further elimination for potential analyses in the future (see Table 19 in the Appendix).

### Eye movement measures

This section presents the description and data for common eye movement measures in the literature, such as word skipping rates, fixation duration, count and location variables, saccadic amplitude, and reading rate. Eye movement measures were either retrieved from the Data Viewer software or calculated using several variables provided by the software. The full set of eye movement variables in TURead is presented in Table  20 in the Appendix. The following sections present a snapshot of the values for selected variables.

#### Word skipping

This section presents the descriptive statistics by condition and by reading modality (oral reading vs. silent reading.) for skipping (Table 6). The stimuli of the present study consisted of 192 texts including one target word each, organized according to their frequencies and lengths in four conditions: Short-Frequent (SF), Short-Infrequent (SI), Long-Frequent (LF), and Long-Infrequent (LI) target words.

In general, the findings show that word skipping is more frequently observed in short words, both in silent reading and oral reading, compared to long words, which is consistent with the findings reported in the literature.

#### Fixation, saccades, and reading rates

In this section, the eye movement measures are presented in terms of six major variables: Fixation Duration (FD) in terms of First Fixation Duration (FFD), Gaze Duration (GD, also known as First Pass Dwell Time), and Total Fixation Duration (TFD); Saccadic Amplitude (Amp) in terms of the Last Saccade (Last) and the Next Saccade (Next); First Pass Fixation Count (FPFC), First Fixation Location (FFL), Launch Site (LS) and Reading Rate (RR) in Table 7. Fixations after the first fixation on the right bottom fixation marker (that is, rereadings) were removed, except for reading rate calculation.

The findings show that the first pass fixation counts (1) increase as the length of words increases, (2) increase as the frequency of words decreases, and (3) are more frequent in oral reading than in silent reading. Another finding is that the mean first fixation and gaze durations are longer for oral reading than for silent reading. Moreover, the mean first landing positions are slightly to the left of the word center, and the saccade amplitude is approximately seven characters for oral reading, whereas it is about eight characters for silent reading. These findings are largely compatible with the literature on reading research in most languages.

### Audio recording analysis and the eye voice span measures

The texts and paragraphs read aloud by participants were recorded as waveform (.wav) audio files, separately for each trial. The start times of the articulation and the end times of the articulation of the target words were manually annotated using the ELAN software (Brugman & Russel, 2008). The beginnings and ends of the articulations were identified listening to the audio files and marking the wave beginnings in the ELAN interface. The tier sets included one tier for each target word imported into the ELAN file (.eaf) for each participant. ELAN annotations were labeled on those tiers. If a target word was not articulated correctly in a trial (e.g., in case of the utterance of a different word than the written one, reading the target word more than once, or stuttering while reading the target word), the audio file of that trial was not annotated and was removed from the analyses. In total, 92.39% of the audio recording annotations were controlled and refined by a second annotator. The annotations provided time stamps of the start and end of an articulation, which allowed synchronization of articulation times and eye movements. For synchronization, the start times of the audio recording and the first fixation start times were calculated according to the eye tracker time using (1) and (2).

1 $$\begin{aligned} A_{tracker} = A_{pc} - t_{pc} + t_{tracker} \end{aligned}$$

2 $$\begin{aligned} FF_{tracker} = FF_{trial} + t_{trial} \end{aligned}$$

The start times for the audio recording were calculated using (1), where A_tracker_ stands for the start time for the audio recording in the eye tracker time. A_pc_ is the start time of the audio recording on the display PC time, which was recorded in a variable defined for this purpose. t_pc_ is the current time of the display PC, recorded in a separate variable. Finally, t_tracker_ is the current eye tracker time when t_pc_ value is updated. The fixation start and end times were provided relative to the trial start time by the Data Viewer software. Therefore, the first fixation start time was calculated relative to the start time of the tracker by (2), where FF_tracker_ is the first fixation start time relative to the start time of the eye tracker, FF_trial_ is the first fixation start time relative to the start time of the trial, and t_trial_ is the start time of the trial relative to the start time of the eye tracker. The variables used to calculate FF_tracker_ were provided by the Data Viewer software.

**Fig. 2:**
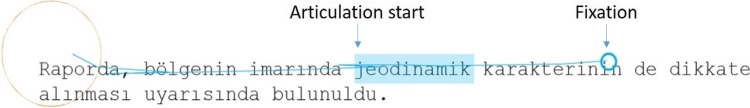
Eye Voice Span (EVS) in character count

Figure 2 shows an example of a fixation immediately following the target word (n+1). In the example, at the start time of the articulation of the target word *jeodinamik* ‘geodynamics’, there is a fixation on the second ‘*i*’ of *karakterinin* ‘of character’ at n+1. Accordingly, the EVS value in this example consists of 22 characters and the value of the EVS-word is 1.

**Table 6:** Number and percentage of skipped and fixated words in oral reading and silent reading, for Short-Frequent (SF), Short-Infrequent (SI), Long-Frequent (LF), and Long-Infrequent (LI) target words

Variables	SF	SI	LF	LI	Total
Oral Reading					
Skipped	447	343	92	82	964
	9.52%	7.30%	1.96%	1.75%	5.14%
Fixated	4247	4353	4598	4608	17806
	90.48%	92.70%	98.04%	98.25%	94.86%
Silent Reading					
Skipped	544	510	94	85	1233
	11.66%	10.95%	2.02%	1.82%	6.61%
Fixated	17413	4122	4146	4568	4577
	88.34%	89.05%	97.98%	98.18%	93.39%

**Table 7:** Fixations, saccades, and reading rates in oral and silent reading (standard deviations in parentheses)

Variables		SF	SI	LF	LI	Mean
Oral Reading						
FD	***FFD***	270.77	302.06	252.0	275.59	274.26
		(111.26)	(140.51)	(100.28)	(129.77)	(121.65)
	***GD***	366.63	481.69	607.96	882.11	568.68
		(171.91)	(251.61)	(258.31)	(394.89)	(328.69)
	***TFD***	417.14	593.93	703.17	1017.76	664.41
		(206.77)	(312.08)	(285.46)	(429.98)	(374.73)
AMP	***Last***	6.84	6.69	8.25	8.08	7.45
		(1.86)	(1.83)	(2.24)	(2.16)	(2.15)
	***Next***	5.68	5.50	6.16	6.21	5.88
		(4.21)	(4.47)	(5.10)	(4.68)	(4.64)
FPFC		1.45	1.77	2.48	3.49	2.24
		(0.67)	(0.99)	(1.06)	(1.54)	(1.31)
FFL		3.59	3.42	4.86	4.67	4.12
		(1.57)	(1.54)	(2.03)	(1.95)	(1.89)
LS		3.25	3.27	3.39	3.41	3.33
		(1.93)	(1.92)	(2.06)	(2.01)	(1.98)
RR		101.74	97.08	95.43	93.18	97.07
		(18.77)	(18.43)	(17.0)	(16.85)	(18.09)
Silent Reading						
FD	***FFD***	232.17	254.89	224.34	249.03	239.96
		(85.55)	(112.31)	(77.8)	(98.75)	(95.09)
	***GD***	285.86	373.79	397.61	674.79	438.23
		(142.85)	(247.81)	(214.18)	(497.75)	(343.73)
	***TFD***	380.72	577.81	528.63	924.87	609.36
		(248.89)	(400.20)	(337.93)	(659.14)	(487.14)
AMP	***Last***	7.86	7.56	9.42	8.93	8.48
		(2.35)	(2.28)	(2.65)	(2.51)	(2.57)
	***Next***	6.16	5.73	7.75	7.69	6.88
		(4.88)	(5.16)	(5.91)	(5.76)	(5.54)
FPFC		1.28	1.52	1.87	2.84	1.90
		(0.56)	(0.89)	(0.91)	(1.92)	(1.34)
FFL		3.76	3.59	5.01	4.81	4.32
		(1.72)	(1.67)	(2.09)	(2.01)	(1.99)
LS		4.10	3.97	4.40	4.13	4.16
		(2.53)	(2.53)	(2.72)	(2.55)	(2.59)
RR		131.79	122.03	128.08	117.18	124.65
		(39.96)	(36.84)	(38.35)	(37.07)	(38.46)

FD: Fixation Duration, FFD: First Fixation Duration (FFD), GD: Gaze Duration (aka. First Pass Dwell Time), TFD: Total Fixation Duration, AMP: Saccadic Amplitude, FPFC: First Pass Fixation Count, FFL: First Fixation Location, LS: Launch Site RR: Reading Rate. Duration values are expressed in milliseconds, amplitude values are expressed in characters, and RR values in wmp (words per minute)

Four Eye Voice Span (EVS) measures were included in TURead: (i) The duration between the beginning of the articulation of a word and the first fixation time on the target word, the Fixation Speech Interval, FSI, following the relevant studies on EVS (Inhoff et al., 2011), (ii) the distance between the first letter of the target word and the character fixated at the beginning of the articulation of the target word, in terms of character count (EVS-char), (iii) the distance between the target word and the word fixated at the beginning of the articulation of the target word, in terms of word count (EVS-word), and (iv) the duration of the articulation.

**Fig. 3:**
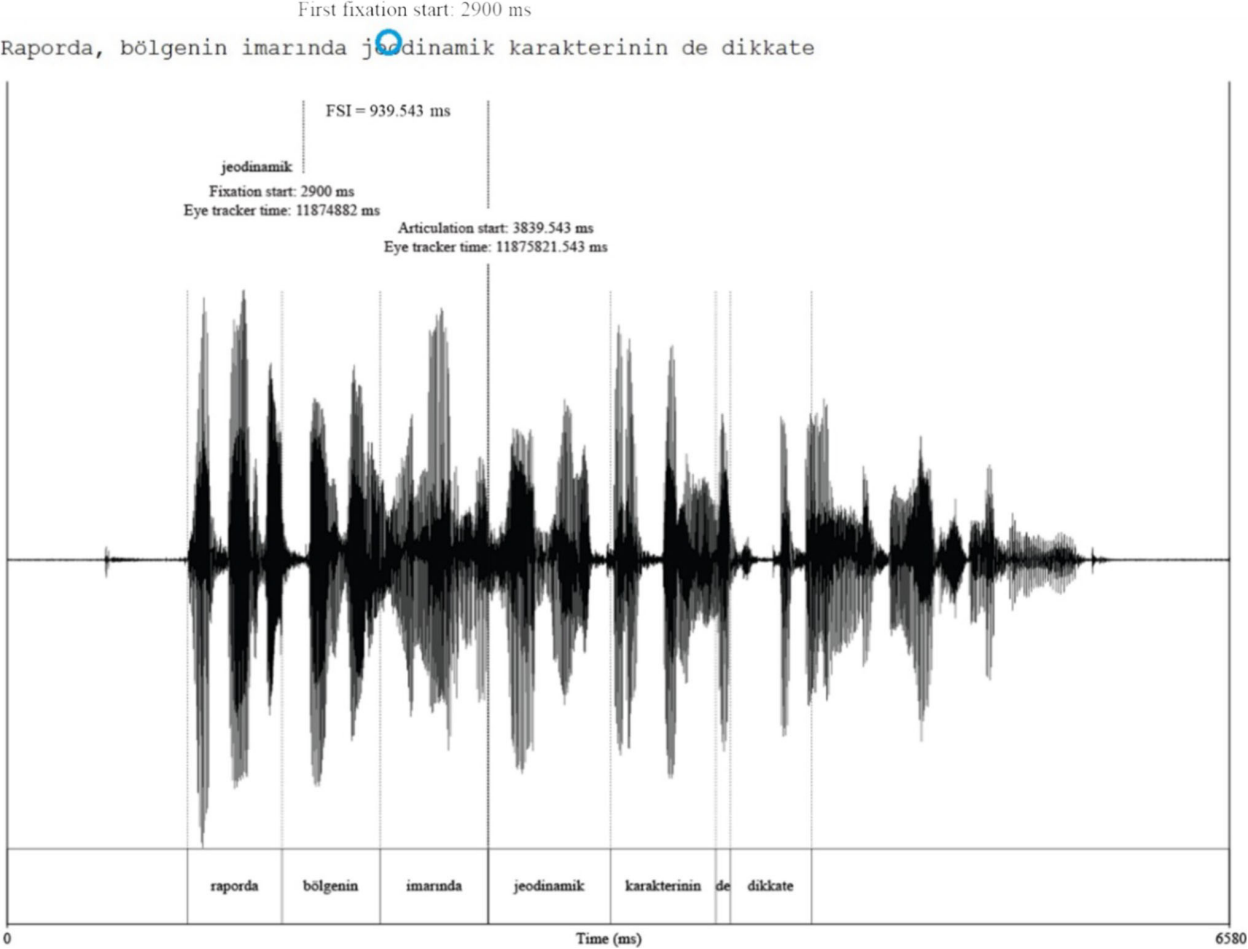
A sample Eye Voice Span (EVS) in time interval (FSI)

A sample FSI (Fixation Speech Interval) is illustrated in Fig. 3. The first fixation on the target word *jeodinamik* ‘geodynamics’ starts 2900 ms after the onset of the trial. The articulation of the same word, in this example, starts 3839.54 ms after the onset of the trial. The resulting FSI is 939.54 ms.

The variables related to oral reading and their descriptions are presented in Table 8.

**Table 8:** Variables related to oral reading

Variable	Description
FSI	The duration between the beginning of the articulation of the target word and the first fixation time on the target word (i.e., fixation speech interval)
EVS-char	The distance between the first letter of the target word and the character that is fixated at the beginning of the articulation of the target word, in terms of character count
EVS-word	The eye voice span in terms of word count
Articulation Duration	The duration of the articulation of the target word

Table 9 shows the values of the EVS measures in TURead, for Short-Infrequent (SI) words, Long-Infrequent (LI) words, Short-Frequent (SF) words, and Long-Frequent (LF) target words.

**Table 9:** Eye Voice Span (EVS) measures in oral reading

Variables	SF	SI	LF	LI	Mean
FSI (ms)	688.27	786.70	699.64	798.12	738.41
(Fixation Speech Interval)	(326.89)	(364.17)	(323.14)	(314.09)	(336.45)
EVS-char	11.82	9.74	12.20	8.79	10.78
	(3.32)	(3.62)	(3.99)	(3.62)	(3.91)
EVS-word	1.02	0.80	0.38	0.17	0.60
	(0.46)	(0.48)	(0.49)	(0.35)	(0.57)
Articulation	390.95	453.75	687.75	807.33	574.95
Duration (ms)	(111.74)	(143.28)	(148.20)	(201.94)	(224.83)

The findings show that the mean FSI values are higher in oral Turkish reading compared to English and German under all conditions (486 ms for English Inhoff et al., 2011), 561 ms for German Laubrock & Kliegl 2015). However, the values are close to the FSI values reported for Finnish (625 ms, Järvilehto et al., 2009). Given that Turkish and Finnish share agglutinating characteristics and shallow orthograpies, the findings are not unexpected. However, the mean values of EVS-char (i.e., the spatial measure of EVS in terms of character count) are shorter in all four conditions compared to the findings reported in the literature (e.g., 15-17 characters in Buswell (1920); 16 characters calculated from the first fixation onset in Laubrock & Kliegl (2015)). In Turkish, approximately one more word was viewed during the FSI of short words, while the eyes tended to be on the same word at the beginning of its articulation for long words. We suggest that the discrepancy observed between the EVS measures obtained for Turkish sentence reading and those reported in the literature (except those for Finnish) is a result of the shallow orthography of Turkish. On the other hand, the inflated FSI could be an indicator of increased prelexical phonological processing for languages with shallow orthographies, as suggested in Frost (1998, 2005). However, these claims require further investigation with cross-linguistic studies.

### Prelexical characteristics

A set of prelexical characteristics were included in TURead, which identified the characteristics of a target word (n), the word prior to the target word (n-1), and the word next to the target word (n+1), including Vowel Harmony, and a set of variables for bigrams and trigrams. These were selected due to the potential impact of the phonological characteristics of Turkish words, particularly vowels.

The Turkish alphabet includes eight vowels, grouped according to the height of the tongue, the roundedness of the lips, and the frontness of the tongue during articulation (Table 10).

**Table 10:** Vowels in Turkish

	Rounded		Unrounded	
	Front	Back	Front	Back
High	ü	u	i	ı
Non-high	ö	o	e	a

Vowel distribution in Turkish words is mostly restricted according to vowel harmony rules. The vowels in the suffixes usually agree with the vowel in the last syllable of the stem to preserve vowel harmony, although there are exceptions (e.g., *-ki*, one of the frequently used suffixes, also used in the present study). Most of the exceptions to vowel harmony in Turkish are loan words (Göksel & Kerslake, 2005). The vowel sequences, allowed according to vowel harmony, are presented in Table 11.

**Table 11:** Vowel sequences allowed according to vowel harmony

	Vowel of the syllable	Vowel allowed in the next syllable
Back	Unrounded (a, ı)	Unrounded (a, ı)
	Rounded (o, u)	Unrounded and Non-high (a)
		Rounded and High (u)
Front	Unrounded (e, i)	Unrounded (e, i)
	Rounded (ö, ü)	Unrounded and Non-high (e)
		Rounded and High (ü)

The variable VH (Vowel Harmony) was included in TURead as a categorical variable with two levels, showing whether the rule was broken or not. A respected VH rule was labeled *0*, and a broken VH rule was labeled *1*. In addition, the number of broken instances was calculated, as presented in the next section.

Further characteristics considered in designing TURead were Trigram Frequency (TF) and Bigram Frequency (BF) of n (the target word), n-1 (the word preceding the target word), and n+1 (the word following the target word). They are assumed to capture the phoneme environment since different pronunciations of phonemes (i.e., allophones) are context dependent. For instance, /h/ is pronounced as a voiceless palatal fricative when it precedes a front vowel. It is also pronounced as a voiceless velar fricative when a back vowel precedes it or as a voiceless glottal fricative when it precedes a back vowel. Sometimes, when it occurs between two identical vowels, it is silent (Göksel & Kerslake, 2005). Due to the restrictions of letter clusters at word-initial and word-final positions, trigrams and bigrams were divided into three subgroups: word-initial, word-final, and between these two. For each group, the frequency values were obtained separately from the BOUN corpus (Sak et al., 2008), depending on the place of the trigram/bigram in the word (Bilgin, 2016). Each prelexical frequency values were calculated as occurrences per million, using Laplace smoothing applying (3), following the previous work on the topic (Brysbaert & Diependaele, 2013) since the data included zero frequency values.

3 $$\begin{aligned} Fpm = ((Count + 1) / (Token + Type)) * 1,000,000 \end{aligned}$$

In (3), *Fpm* stands for frequency per million, *Count* stands for the number of occurrences of a word in the corpus, *Token* stands for the number of word tokens in the corpus, and *Type* stands for the number of word types in the corpus. The average adjacent trigram and adjacent bigram frequencies per million were included in the dataset as trigram frequency (TF) and bigram frequency (BF), respectively. Another important restriction regarding word boundaries was captured by inclusion of the average of word-initial and word-final trigram/bigram frequencies within calculations. The variables for prelexical characteristics and their descriptions are provided in Table 21 in the Appendix, and the number of *types* and *tokens* used in (3) are provided in Table 12.

**Table 12:** The number of types and tokens used for trigram and bigram calculation

	Position in the word	Number of types	Number of tokens
Trigram	Word initial	4,041	354,203,718
	Anywhere	9,241	1,728,651,926
	Word final	4,276	354,203,718
Bigram	Word initial	514	383,224,629
	Anywhere	790	2,110,491,073
	Word final	560	381,839,147

The descriptive statistics for the trigram and bigram frequencies and the number of broken vowel harmony instances are presented in the following section, together with the predictability scores and lexical characteristics of the words.

**Table 13:** Characteristics of the target word (n)

Variables		SF	SI	LF	LI	Mean
Oral Reading						
Word Pred.	***122p***	0.02	0.01	0.00	0.00	0.01
		(0.07)	(0.09)	(0.03)	(0.00)	(0.06)
	***35p***	0.02	0.01	0.00	0.00	0.01
		(0.08)	(0.08)	(0.03)	(0.00)	(0.06)
Suffix Pred.	***1suffix(110p)***	0.31	0.09	0.26	0.18	0.22
		(0.18)	(0.05)	(0.17)	(0.12)	(0.17)
	***1suffix(35p)***	0.34	0.11	0.27	0.20	0.23
		(0.20)	(0.06)	(0.18)	(0.12)	(0.18)
	***2suffix(69p)***	0.14	0.05	0.12	0.10	0.10
		(0.08)	(0.04)	(0.07)	(0.06)	(0.07)
	***2suffix(35p)***	0.13	0.04	0.13	0.09	0.10
		(0.06)	(0.03)	(0.08)	(0.06)	(0.07)
Familiarity		6.96	2.89	6.95	4.67	5.48
		(0.36)	(2.33)	(0.33)	(2.53)	(2.39)
Length	***Word***	6.14	6.03	11.95	11.87	8.91
		(1.60)	(1.62)	(1.64)	(1.64)	(3.33)
	***Stem***	4	4	10	10	6.91
		(0)	(0)	(0)	(0)	(3)
Suffix Count		1.07	1.02	0.98	0.94	1.00
		(0.80)	(0.81)	(0.82)	(0.82)	(0.81)
Frequency	***Word***	0.30	$-$2.18	0.05	$-$1.93	$-$0.84
		(1.04)	(0.63)	(1.30)	(0.79)	(1.50)
	***Stem***	1.32	$-$1.48	1.37	$-$1.00	0.16
		(0.29)	(0.65)	(0.57)	(0.50)	(1.41)
	***Trigram***	2.67	2.60	3.03	2.83	2.78
		(0.51)	(0.82)	(0.24)	(0.31)	(0.55)
	***Bigram***	3.77	3.74	3.85	3.78	3.79
		(0.27)	(0.34)	(0.13)	(0.14)	(0.24)
Broken VH count		1998	1452	3084	2593	9127
Silent Reading						
Word Pred.	***122p***	0.02	0.01	0.00	0.00	0.01
		(0.07)	(0.07)	(0.03)	(0.00)	(0.05)
	***35p***	0.02	0.01	0.00	0.00	0.01
		(0.08)	(0.06)	(0.03)	(0.00)	(0.05)
Suffix Pred.	***1suffix(110p)***	0.31	0.09	0.25	0.18	0.21
		(0.19)	(0.05)	(0.17)	(0.11)	(0.16)
	***1suffix(35p)***	0.34	0.11	0.26	0.19	0.23
		(0.20)	(0.06)	(0.18)	(0.12)	(0.17)
	***2suffix(69p)***	0.13	0.06	0.12	0.10	0.10
		(0.08)	(0.04)	(0.07)	(0.06)	(0.07)
	***2suffix(35p)***	0.13	0.05	0.13	0.09	0.10
		(0.06)	(0.04)	(0.08)	(0.06)	(0.07)
Familiarity		6.96	2.79	6.95	4.020	5.19
		(0.36)	(2.29)	(0.33)	(2.63)	(2.53)
Length	***Word***	6.19	6.17	12.0	12	6.19
		(1.60)	(1.60)	(1.63)	(1.63)	(3.33)
	***Stem***	4	4	10	10	7.15
		(0)	(0)	(0)	(0)	(3)
Suffix Count		1.09	1.08	1.00	1.00	1.04
		(0.80)	(0.80)	(0.82)	(0.82)	(0.81)
Frequency	***Word***	0.28	$-$2.23	0.00	$-$2.04	$-$1.00
		(1.04)	(1.04)	(1.31)	(0.75)	(1.50)
	***Stem***	1.32	$-$1.49	1.36	$-$1.09	0.03
		(0.29)	(0.29)	(0.56)	(0.54)	(1.42)
	***Trigram***	2.68	2.64	3.02	2.83	2.80
		(0.51)	(0.78)	(0.24)	(0.30)	(0.52)
	***Bigram***	3.77	3.76	3.85	3.79	3.79
		(0.27)	(0.32)	(0.13)	(0.15)	(0.23)
Broken VH count		2052	1680	3310	3700	10742

The values in parentheses show the standard deviations. The frequency values in the table are log-transformed (base 10). *p*: participants

Table 14 presents the characteristics of the words that precede the target word (n-1). Table 15 presents the characteristics of the words that follow the target word (n+1).

**Table 14:** Characteristics of the words that precede the target word (n-1)

Variables		SF	SI	LF	LI	Mean
Oral Reading						
Word Pred.	***35p***	0.06	0.09	0.05	0.07	0.07
		(0.12)	(0.22)	(0.11)	(0.17)	(0.16)
Length	***Word***	7.87	7.22	8.59	7.67	7.87
		(2.33)	(2.11)	(2.30)	(2.31)	(2.32)
	***Stem***	5.18	4.93	5.23	5.35	5.17
		(1.78)	(2.27)	(1.70)	(1.67)	(1.87)
Suffix Count		1.22	1.04	1.34	1.07	1.18
		(0.92)	(0.88)	(0.93)	(0.80)	(0.90)
Frequency	***Word***	1.33	1.36	1.35	1.25	1.32
		(1.15)	(1.37)	(1.22)	(1.19)	(1.23)
	***Stem***	1.50	1.57	1.43	1.14	1.42
		(1.17)	(1.42)	(1.23)	(1.41)	(1.31)
	***Trigram***	3.35	3.21	3.39	3.25	3.30
		(0.30)	(0.46)	(0.34)	(0.34)	(0.37)
	***Bigram***	3.96	3.93	4.00	3.95	3.96
		(0.23)	(0.26)	(0.16)	(0.21)	(0.22)
Broken VH count		803	416	920	767	2906
Silent Reading						
Word Pred.	***35p***	0.06	0.08	0.05	0.07	0.06
		(0.12)	(0.21)	(0.10)	(0.17)	(0.16)
Length	***Word***	7.88	7.20	8.56	7.72	7.85
		(2.32)	(2.11)	(2.31)	(2.38)	(2.34)
	***Stem***	5.21	4.94	5.28	5.27	5.18
		(1.78)	(2.26)	(1.68)	(1.72)	(1.87)
Suffix Count		1.22	1.04	1.32	1.14	1.18
		(0.92)	(0.88)	(0.94)	(0.84)	(0.90)
Frequency	***Word***	1.35	1.36	1.37	1.29	1.34
		(1.13)	(1.37)	(1.20)	(1.22)	(1.23)
	***Stem***	1.54	1.54	1.46	1.16	1.42
		(1.14)	(1.43)	(1.23)	(1.41)	(1.32)
	***Trigram***	3.35	3.19	3.38	3.25	3.30
		(0.30)	(0.49)	(0.34)	(0.32)	(0.37)
	***Bigram***	3.96	3.92	4.00	3.96	3.96
		(0.23)	(0.26)	(0.16)	(0.21)	(0.22)
Broken VH count		809	442	1054	1124	3429

The values in parentheses show the standard deviations. The frequency values in the table are log-transformed (base 10)

**Table 15:** The characteristics of the words that follow the target word (n+1)

Variables		SF	SI	LF	LI	Mean
Oral Reading						
Word Pred.	***35p***	0.05	0.02	0.02	0.02	0.03
		(0.14)	(0.04)	(0.06)	(0.04)	(0.08)
Length	***Word***	8.09	8.32	8.66	8.04	8.29
		(2.38)	(3.39)	(1.93)	(2.38)	(2.58)
	***Stem***	5.63	5.57	5.78	5.47	5.62
		(1.50)	(2.25)	(1.89)	(1.70)	(1.86)
Suffix Count		1.16	1.18	1.25	1.15	1.19
		(1.11)	(0.97)	(1.04)	(0.99)	(1.03)
Frequency	***Word***	1.20	0.92	1.28	1.48	1.21
		(1.36)	(1.29)	(0.83)	(1.20)	(1.20)
	***Stem***	1.37	1.02	1.61	1.64	1.41
		(1.22)	(1.22)	(1.05)	(1.07)	(1.17)
	***Trigram***	3.24	3.14	3.37	3.30	3.26
		(0.45)	(0.55)	(0.35)	(0.37)	(0.45)
	***Bigram***	3.89	3.92	3.95	3.92	3.92
		(0.25)	(0.25)	(0.18)	(0.19)	(0.22)
Broken VH count		1101	819	928	538	3386
Silent Reading						
Word Pred.	***35p***	0.05	0.02	0.02	0.02	0.03
		(0.13)	(0.04)	(0.06)	(0.04)	(0.08)
Length	***Word***	8.09	8.34	8.66	8.00	8.27
		(2.41)	(3.40)	(1.94)	(2.64)	(2.65)
	***Stem***	5.61	5.59	5.79	5.44	5.61
		(1.50)	(2.26)	(1.89)	(1.81)	(1.89)
Suffix Count		1.16	1.18	1.24	1.13	1.18
		(1.12)	(0.97)	(1.05)	(1.02)	(1.04)
Frequency	***Word***	1.15	0.87	1.27	1.48	1.20
		(1.40)	(1.32)	(0.84)	(1.26)	(1.23)
	***Stem***	1.35	1.00	1.60	1.66	1.41
		(1.25)	(1.23)	(1.06)	(1.05)	(1.17)
	***Trigram***	3.22	3.14	3.37	3.29	3.26
		(0.47)	(0.55)	(0.36)	(0.38)	(0.45)
	***Bigram***	3.89	3.91	3.95	3.92	3.92
		(0.25)	(0.25)	(0.18)	(0.21)	(0.22)
Broken VH count		1088	878	1052	856	3874

The values in parentheses show the standard deviations. The frequency values in the table are log-transformed (base 10)

### Predictability scores and lexical characteristics

The predictability scores were collected from 122 participants for the target words, 70 participants for the neighboring words (35 for n-1 and 35 for n+1), 110 participants for the suffix of one-suffixed target words and 69 participants for the suffixes of two-suffixed target words. The least data were collected for neighboring words (35 participants). To have balanced data from the participants for analyses that require it, a randomly selected sample set of 35 participant scores were included for target words and suffixes. The predictability scores of 192 target words from 122 participants and that of the selected 35 participants (*M* = 23.66, *SD* = 3.89 years old, three participants did not report birth year; 35 females) were not significantly different (*F*(1,382) = 0.00004, *p* $=.995$), which justified the selection of a smaller set as a representative set for the predictability scores. The prediction of the suffix of one-suffixed target words from 110 participants and that of selected 35 participants (*M* = 22.06, *SD* = 3.32 years old, four participants did not report birth year; 15 females, one participant did not report any gender) were not significantly different (*F*(1,126) = 0.366, *p* $=.546$), and neither were the prediction of the suffixes of two-suffixed target words from 69 participants and that of selected 35 participants (*M* = 21.66, *SD* = 1.54 years old, three participants did not report birth year; 15 females) (*F*(1,126) = 0.05, *p* $=.824$). In addition to the randomly selected sample sets of 35 participant scores for target words and suffixes, predictability scores of all available data were also included in the TURead Dataset. The information of the number of participants that contributed to the predictability scores for each predictability variable in the dataset was indicated in the variable name. For example, there are two variables for word (n) predictability scores such that the variable named p0_122_participants is calculated on the scores of 122 participants and the variable named p0_35_participants is calculated over the scores of 35 participants. For all predictability data, the correct predictions were scored as *1*, and the incorrect predictions were scored as *0*. The probability (*p*) of a correct prediction was calculated using (4), where *num* stands for the number of predictions for each word.

4 $$\begin{aligned} p = number\ of\ correct\ predictions\ /\ num \end{aligned}$$

The variables for the predictability calculations and their descriptions are presented in Table 22 in the Appendix.

In addition to word-level and suffix-level predictability, TURead includes further variables that identify the characteristics of a target word (n), the word prior to the target word (n-1), and the word next to the target word (n+1), such as familiarity ratings, word lengths, inflectional suffix counts, stem lengths, word frequencies, stem frequencies, trigram and bigram frequencies, and vowel harmony states.

Surface frequency values were obtained from the BOUN corpus (Sak et al., 2008). Lexical frequencies per million were calculated using Laplace smoothing (Brysbaert & Diependaele, 2013) applying (3). The number of word tokens was 383,224,629 and the number of word types was 1,337,898 used for the calculation. The *Fpm* values for lexical frequencies can be back-transformed using the same formula. The variables for the lexical characteristics of the words and their descriptions are presented in Table 23 in the Appendix. Table 13 presents the characteristics of the target words (n).

## Discussion

The present study presents TURead, an eye movement dataset of silent and oral reading in Turkish, with an experimental approach in which the target words were manipulated on the basis of word length, frequency, and number of suffixes. TURead aims to provide empirical data for a diverse set of analyses. To provide benchmark data for analyses, word characteristics variables such as length, frequency, and predictability of target words and neighboring words are provided in the dataset (e.g., Kliegl et al., 2006). For further analysis of the influences of morphological complexity and phonological processing on eye movements during reading, a set of variables related to morphological complexity (e.g., suffix counts, suffix predictabilities, and stem lengths and frequencies) and prelexical characteristics of target words were also included in TURead to address the potential impact of phonotactics in Turkish (Acartürk et al., 2017). Furthermore, the familiarity scores of the target words included in TURead provide data to further investigations of strong vs. weak approaches to early phonological processing (Frost 1998, 2005, Coltheart et al., 2001; e.g., Özkan 2020). Turkish, as an agglutinating language with rich morphology and shallow orthography, provides a suitable environment for such investigations.

In addition to mostly studied eye movement measures (e.g., first fixation duration, gaze duration, last saccade amplitude, next saccade amplitude, first fixation location and launch site) for both oral and silent reading, four specific measures of oral reading were included in TURead (i.e., fixation speech interval, eye-voice span in terms of character count, eye-voice span in terms of word count and duration of the articulation). As previous research indicates, the eye-voice span measures reflect the manageable count of items held in the memory buffer during reading (e.g., Laubrock & Kliegl 2015; Inhoff et al., 2011). Together with working memory test scores (i.e., Corsi Block test and digit span test scores), oral reading-specific variables provided in TURead could be used in analyses that address memory processes and post-lexical processing involved in reading (e.g., Özkan 2020).

## Conclusion

This study presented a new eye movement dataset of sentence reading in Turkish. The dataset consists of 192 sentences read by 215 participants in silent and oral modalities. The variables in the dataset were described together with the data collection, data cleaning, and data analysis procedures. The descriptive statistics of the selected variables in both reading modalities were prelexical and lexical characteristics of previous (n-1), target (n), and next words (n+1), and familiarity ratings of words. In addition to the descriptive statistics of the selected oculomotor measures such as fixation durations and saccade amplitudes, we also reported our findings on FSI (fixation speech interval) and EVS (eye voice span) in Turkish reading. We observed that FSI in Turkish is greater than in English and German but close to Finnish, which also has a shallow orthography. The increase in FSI in languages such as Turkish and Finnish may point to the influence of shallow orthography on prelexical phonological processing. We also observed shorter EVS values in Turkish sentence reading compared to previous research. Again, this difference may be explained by the effect of shallow orthography on the working memory buffer (Laubrock & Kliegl, 2015). More studies are needed in Turkish and other languages with shallow orthography to provide more evidence to support our findings. We believe that TURead will be a valuable and helpful resource for researchers to investigate the interplay between language characteristics and eye movements during reading.

## Data Availability

The files that include datasets, stimulus texts, and variable explanations can be downloaded from *TURead: An Eye Movement Dataset of Turkish Reading* in Open Science Framework OSF Repository, under the folder *TURead_files* (TURead link: https://osf.io/w53cz/). TURead dataset is provided as an Excel file, TURead_target_words.xlsx. An additional data set that includes eye movement measures of both oral and silent reading, and lexical and prelexical characteristics (i.e., word lengths, inflectional suffix counts, stem lengths, word frequencies, stem frequencies, trigram and bigram frequencies, and vowel harmony states) for all words was provided for further analyses in another Excel file, TURead_all_words.xlsx. The variables for target words and other words were combined in one Excel file, TURead_variables.xlsx, on separate sheets. None of the experiments reported here was preregistered.

## Appendix

The variable explanations provided in this section belong to the variables in the target word dataset. The variables in the additional dataset for all words are provided on the relevant sheet of the file, TURead_variables.xlsx which can be downloaded from *TURead: An Eye Movement Dataset of Turkish Reading* in Open Science Framework OSF Repository, under the folder *TURead_files*.^5^

**Table 16:** General variables common to the participants and the stimuli

Variable	Description
PARTICIPANT	The ID number of each participant
RECORDING SESSION LABEL	The ID number of each part of an experiment session organized as participant code + part information (p1: part one, p2: part two)
TRIAL INDEX	The order of one trial within each part of an experiment session
TARGET WORD	The original target word of stimuli texts
TARGET WORD WITHOUT TURKISH CHARACTERS	Target words in which Turkish characters were replaced such that ç replaced by c, ı replaced by i, ğ replaced by g, ö replaced by o, ş replaced by s, ü replaced by u
WORD CODE W0	The unique code for W0 (i.e., the target word / word n)
WORD CODE W1	The unique code for W1 (i.e., word n-1)
WORD CODE W2	The unique code for W2 (i.e., word n+1)
READING MODALITY	The modality of reading (oral vs. silent)
CONDITION	The condition set for the target word. SI: Short-Infrequent words, LI: Long-Infrequent words, SF: Short-Frequent words, and LF: Long-Frequent words
IA ID	The order of the target word within the text, by the software as the ordinal ID of the current interest area
W1 IA ID	The order of the word on the left of the target word (word n-1) within the text, by the software as the ordinal ID of the current interest area
W2 IA ID	The order of the word on the left of the target word (word n+1) within the text, by the software as the ordinal ID of the current interest area

**Table 17:** Variables for further analyses (Part 1)

Variable	Description
ARTICULATION OF ONSET FIXATION IA ID	The order of the word within the text that was fixated at the onset of the articulation of the target word
ARTICULATION ONSET FIXATION DURATION	The duration of the fixation at the onset of the articulation of the target word
ARTICULATION START ET	The beginning of the articulation of the target word according to the eye tracker time
ARTICULATION END ET	The end of the articulation of the target word according to the eye tracker time
FFIX ET TIME	The beginning of the first fixation on the target word relative to the eye tracker start time
IA FIRST FIXATION TIME	The beginning of the first fixation on the target word relative to the beginning of the trial (i.e., TRIAL START TIME)
TRIAL START TIME	The start time of the trial since the tracker was activated
IA SECOND FIXATION X	The horizontal position of the second fixation in pixels
IA THIRD FIXATION_X	The horizontal position of the third fixation in pixels
IA FIRST FIXATION_X	The horizontal position of the first fixation in pixels
IA FIRST FIXATION INDEX	The order of the first fixation on the target word
IA FIRST RUN LAST FIX INDEX	The order of the last fixation on the target word in the first pass
IA FIRST RUN LAST FIX X	The horizontal position of the last fixation on the target word in the first pass
IA FIRST RUN NEXT FIX OF LAST FIX IA ID	The order of the word within the text that was fixated immediately after the last fixation on the target word in the first pass
IA FIRST RUN NEXT FIX OF LAST FIX X	The horizontal position of the fixation that was made immediately after the last fixation on the target word in the first pass
IA FIRST RUN PREVIOUS FIX OF FIRST FIX IA ID	The order of the word within the text that was fixated prior to the first fixation on the target word
IA FIRST RUN PREVIOUS FIX OF FIRST FIX X	The horizontal position of the fixation that was made prior to the first fixation on the target word in the first pass
IS FIRST RUN NEXT FIX OF LAST FIX IA BOTTOM	The vertical position of the bottom edge of the interest area of the word that was fixated immediately after the last fixation on the target word in the first pass
IA FIRST RUN PREVIOUS FIX OF FIRST FIX IA BOTTOM	The vertical position of the bottom edge of the interest area of the word that was fixated prior to the first fixation on the target word in the first pass

**Table 18:** The variables for further analyses (cont’d)

Variable	Description
TARGET IA BOTTOM	The vertical position of the bottom edge of the interest area (IA) of the target word
TARGET IA LEFT	The horizontal position of the left edge of the IA of the target word
TARGET IA RIGHT	The horizontal position of the right edge of the IA of the target word
TARGET IA TOP	The vertical position of the top edge of the IA of the target word
AUDIO RECORDING START TIME	The beginning of the audio recording according to the display PC clock. The variable was used to retrieve the time when the audio recording starts for synchronizing eye movements with audio recordings
CURRENT DISPLAY PC TIME	The current time on the display PC clock when the CURRENT EYE TRACKER TIME value is updated. The variable was used for synchronizing eye movements with audio recordings
CURRENT EYE TRACKER TIME	The current time on the eye tracker clock. The variable was used for synchronizing eye movements with audio recordings
DISPLAY ONSET TIME	The onset time of the stimulus according to the display PC clock
IP END TIME	The end time of the interest period. Interest period is the period between the stimulus appearance and disappearance
DISPLAY ONSET ET	The onset time of the stimulus according to the eye tracker time
DURATION TO CHANGE SCREEN	The duration that was required for the change of the screen during a trial which is 1000 ms. That was set by an eye contingent IA for a fixation marker at the right-bottom of the screen appeared together with the stimulus
READING TIME MIN	The reading duration of the stimulus text in minutes. It was calculated by the subtraction of DURATION TO CHANGE SCREEN from the time interval between DISPLAY ONSET ET and IP END TIME
WORD COUNT	The number of words in the stimuli texts

**Table 19:** Data labeling for further elimination in future analyses

Variable	Description
Incorrectly read target	Incorrect articulation of the target word. 1 indicates an error (EVS not calculated)
Fixation and target not on the same line	Whether the word that was fixated at the onset of the articulation of the target word was on the same line as the target word. 1 indicates different lines for which the EVS values were not calculated
Articulation onset fixation not on a word	Whether the fixation that was made at the onset of the articulation of the target word was on a word. 1 if the fixation was not on a word for which the EVS values were not calculated
Negative EVS char	Whether the fixation that was made at the onset of the articulation of the target word was located on a character on the left side of the target word (i.e., Negative EVS char values). 1 indicates negative values, 0 indicates positive values
Negative EVS word	Whether the fixation that was made at the onset of the articulation of the target word was located on a word on the left side of the target word (i.e., Negative EVS word values). 1 indicates negative values, 0 indicates positive values
Negative FSI	Whether the fixation that was made at the onset of the articulation of the target word was before the first fixation on the target word (i.e., Negative FSI values). 1 indicates negative values, 0 indicates positive values
IA first run next fix of last fix not on same line	Whether the word that was fixated immediately after the last fixation on the target word in the first pass (used for OSA calculations) was on the same line as the target word. 1 indicates different lines for which OSA was not calculated
IA first run previous fix of first fix not on same line	Whether the word that was fixated prior to the first fixation on the target word (used for ISA and LS calculations) was on the same line as the target word. 1 indicates different lines for which ISA was not calculated

**Table 20:** Eye movement variables

Variable	Description
IA SKIP	Skipped words marked as 1, by the software
IA FIRST FIXATION DURATION	The duration of the first fixation on the word in the first pass, without considering whether a higher-ID interest area (IA) at the right was fixated before, by the software
IA FIRST RUN DWELL TIME	The sum of the fixation durations on the word in the first pass, without considering whether a higher-ID IA was fixated before, by the software
IA FIRST RUN FIXATION COUNT	The number of fixations on the word in the first pass, without considering whether a higher-ID IA was fixated before, by the software
IA FIRST RUN LANDING POSITION	The first landing position on the word, by the software
IA FIRST RUN LAUNCH SITE	The distance of the fixation preceding the first fixation to the word (launch site), by the software
IA FIRST RUN LANDING POSITION IN CHARACTER COUNT	The first landing position on the word in terms of character count (the space preceding the word as 1), calculated manually
IA FIRST RUN LAUNCH SITE IN CHARACTER COUNT	The distance between the previous fixation to the word in terms of character count (the space preceding the word as 1), calculated manually
OSA	The distance between the last and fixation on the word (outgoing saccade amplitude)
OSA IN CHARACTER COUNT	The distance between the last and next fixation on the word in character count, calculated manually
ISA	The distance between the preceding and current fixation on the word (incoming saccade amplitude)
ISA IN CHARACTER COUNT	The distance between the preceding and current fixation on the word in character count, calculated manually
IA SECOND FIXATION DURATION	The duration of the second fixation on the word, by the software
IA SECOND FIXATION RUN	The order of the run in which the second fixation was made (1 indicates it was made before leaving the word), by the software
IA THIRD FIXATION DURATION	The duration of the third fixation on the word, by the software
IA THIRD FIXATION RUN	The order of the run in which the third fixation was made (1 indicates it was made before leaving the word), by the software
TOTAL FIXATION DURATION	The sum of the fixation durations on the word, calculated manually by removing fixations after the first fixation on the marker
IA REGRESSION OUT	Whether regression(s) were made from the current IA to earlier IAs prior to leaving it in a forward direction. 1 if there is at least one regressive saccade from the IA, 0 otherwise, by the software
IA_REGRESSION _OUT_COUNT	The number of regressions from the current IA to earlier IAs prior to leaving that IA in a forward direction, by the software
IA REGRESSION PATH DURATION	The sum of fixation durations when the current IA is first fixated until the eyes leave the IA in a forward direction, by the software
REGRESSION IN COUNT	The number of times IA was entered from a higher-ID IA. Calculated manually by removing the fixations made after the first fixation on the marker
REGRESSION IN	Whether the current IA received at least one regression from higher-ID IAs. 1 if there is at least one regressive saccade from the IA, 0 otherwise, calculated manually by removing the fixations made after the first fixation on the marker
WPM	Reading rate in words per minute

**Table 21:** The variables for bigrams and trigrams

Variable	Description
TF0 pm	The mean trigram frequency per million* of the target word
BF0 pm	The mean bigram frequency per million* of the target word
VH0	Whether the target word satisfies the vowel harmony rule. 1 indicates a broken rule
TF1 pm	The mean trigram frequency per million* of the word n-1
BF1 pm	The mean bigram frequency per million* of the word n-1
VH1	Whether the word n-1 satisfies the vowel harmony rule. 1 indicates a broken rule
TF2 pm	The mean trigram frequency per million* of the word n+1
BF2 pm	The mean bigram frequency per million* of the word n+1
VH2	Whether the word n+1 satisfies the vowel harmony rule. 1 indicates a broken rule

*The frequency per million values are the transformed values according to the Laplace smoothing method (Brysbaert & Diependaele, 2013)

**Table 22:** The variables for the predictability calculations

Variable	Description
p0 (122 participants)	The probability of the correct prediction of the target word collected from 122 participants
p0 (35 participants)	The probability of the correct prediction of the target word collected from 35 participants
p1 (35 participants)	The probability of the correct prediction of word n-1, collected from 35 participants
p2 (35 participants)	The probability of the correct prediction of word n+1, collected from 35 participants
p1Suffix (110 participants)	The probability of the correct prediction of the suffix of one-suffixed target words, collected from 110 participants
p2Suffix (69 participants)	The probability of the correct prediction of the suffixes of two-suffixed target words, collected from 69 participants
p1Suffix (35 participants)	The probability of the correct prediction of the suffix of one-suffixed target words, collected from 35 participants (i.e., a randomly selected sample set of 35 participants out of 110 participants that provided data for p1suffix)
p2Suffix (35 participants)	The probability of the correct prediction of the suffixes of two-suffixed target words, collected from 35 participants(i.e., a randomly selected sample set of 35 participants out of 69 participants that provided data for p2suffix)

**Table 23:** The variables that identify the lexical characteristics of the words

Variable	Description
WL0.raw	The length of the target word in terms of character count
SC0	The count of the inflectional suffixes of the target word
SL0.raw	The length of the target stem in terms of character count
SF0 pm	The surface frequency per million* of the target stem
WF0 pm	The surface frequency per million* of the target word
WL1 raw	The length of the word n-1 in terms of character count
SC1	The count of the inflectional suffixes of the word n-1
SL1.raw	The length of the stem of word n-1 in terms of character count
SF1 pm	The surface frequency per million* of the stem of word n-1
WF1 pm	The surface frequency per million* of the word n-1
WL2 raw	The length of the word n+1 in terms of character count
SC2	The count of the inflectional suffixes of the word n+1
SL2.raw	The length of the stem of word n+1 in terms of character count
SF2 pm	The surface frequency per million* of the stem of word n+1
WF2 pm	The surface frequency per million* of the word n+1

*The frequency per million values are the transformed values according to the Laplace smoothing method (Brysbaert & Diependaele, 2013)

## Footnotes

1 Although some suffixed frequent words had frequency values below the cut-off point of stem frequencies, the mean frequency values were still homogeneous within a condition.

2 In linguistic analyses of Turkish, capital letters in affixes indicate phonetic variability in line with vowel and/or consonant harmony rules of the language.

3 TURead: An Eye Movement Dataset of Turkish Reading in Open Science Framework OSF Repository, https://osf.io/w53cz/

4 The eye movement data inspection and analyses were started using Data Viewer version 2.3.22. During the analyses, the software was updated to versions 2.6.1 and 3.2.1.
